# Friends or foes: Unraveling the tsetse fly-*Spiroplasma* symbiosis

**DOI:** 10.1371/journal.pntd.0014698

**Published:** 2026-09-10

**Authors:** Serap Aksoy, Brian L. Weiss, Daniel J. Bruzzese, Fabian Gstöttenmayer, Richard Echodu, Giulia Fiorenza, Riccardo Piccinno, Anna Rodolfa Malacrida, Adly M. M. Abd-Alla

**Affiliations:** 1 Department of Epidemiology of Microbial Diseases, Yale School of Public Health, New Haven, Connecticut, United States of America; 2 Gulu University Multifunctional Research Laboratories, Gulu, Uganda; 3 Department of Biology and Biotechnology “Lazzaro Spallanzani”, University of Pavia, Pavia, Italy; 4 Insect Pest Control Laboratory, Joint FAO/IAEA Centre of Nuclear Techniques in Food and Agriculture, Vienna, Austria; University of Pretoria, SOUTH AFRICA

## Abstract

Tsetse flies (*Glossina* spp.) transmit African trypanosomes, the causative agents of human African and African animal trypanosomiases (HAT and AAT, respectively). These neglected tropical diseases impose significant public health and economic burdens across sub-Saharan Africa. Trypanosome transmission by tsetse flies is influenced by multiple factors, including host genetic background, ecological factors, and interactions with heritable microbial endosymbionts. *Spiroplasma glossinidia* has recently emerged as an important modulator of tsetse reproductive fitness and vector competence, making it a potential target for symbiont-based vector control strategies. In this review, we summarize the current knowledge of the tsetse-*Spiroplasma* symbiosis. We detail *Spiroplasma’s* spatial and temporal infection dynamics in laboratory-reared and natural populations. Additionally, we highlight key aspects of the bacterium’s genomics, phylogenetics, and physiological interactions with its tsetse host, including influences on host gene expression reproductive physiology, and vector competence. Finally, we discuss how the tsetse–*Spiroplasma* symbiosis could be harnessed to develop innovative, biological-based vector control and trypanosome transmission-blocking strategies, and we identify critical gaps that must be addressed to translate these findings into effective disease control interventions.

## Introduction

Tsetse flies (*Glossina* spp.) are obligate, cyclic vectors of African trypanosomes, the causative agents of human African trypanosomiasis (HAT) and African animal trypanosomosis (AAT) across much of sub-Saharan Africa. Active disease surveillance followed by treatment of infected hosts has virtually eliminated chronic HAT (caused by *Trypanosoma brucei gambiense*) in west and central Africa. However, sporadic cases of acute HAT (caused by *Trypanosoma brucei rhodesiense*) still appear in East Africa, and AAT continues to pose significant constraints on livestock productivity [1,2]. In the absence of effective vaccines to protect animal hosts, vector control remains the predominant strategy for AAT management [2–4]. Vector control relies on the use of traps and targets, as well as implementation of Sterile Insect and Sequential Aerosol Techniques (SIT and SAT, respectively) in frame with an area-wise integrated pest management (AW-IPM) approach [4], all of which can be improved through research into key aspects of tsetse physiology.

Tsetse flies exhibit polyandric viviparous reproductive biology. Females ovulate a single oocyte per gonotrophic cycle and use the spermatozoa stored in the spermatheca for successive reproductive cycles. Fertilization, embryogenesis, and larval development occur entirely *in utero*, and females produce one offspring per 10-day gonotrophic cycle. Developing larvae are nourished by maternal milk gland secretions and are deposited as fully developed third-instar larvae that rapidly burrow into the surrounding substrate (e.g., soil or leaf litter) and pupate [5]. Hence, vector control methods that reduce tsetse population size by traps and targets or impair its reproductive output (SIT) are highly effective at reducing disease transmission. In addition to vector population reduction, approaches that could interfere with parasite development within the tsetse host can reduce vector competence and disease transmission. It has not been possible to develop transgenic tsetse due to the fly’s peculiar reproductive biology [5], but endosymbiotic microbes that reside in tsetse’s gut provide an opportunity to employ paratransgenic methods to modify vector competence [6].

**Fig 1:**
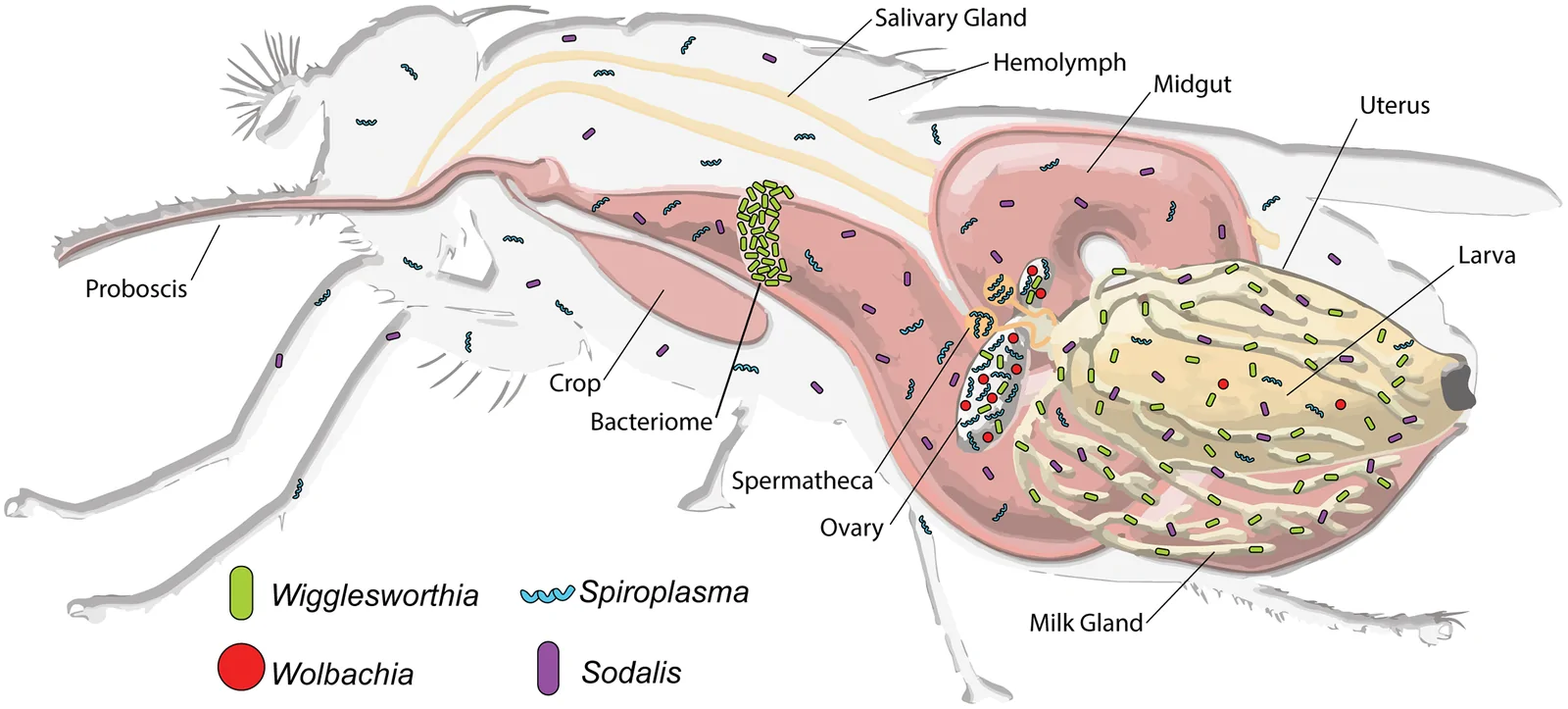
A diagram of an adult tsetse female with a fully developed larva in its uterus. The different colors denote the four endosymbionts inhabiting different tissues. The green cylinders indicate the location of intracellular *Wigglesworthia* in the midgut bacteriome organ as well as extracellular forms residing within the milk gland lumen, which are present in all tsetse species. The red dots indicate *Wolbachia* infections that primarily inhabit the reproductive organs, preferentially in the *morsitans* group of tsetse species. The purple cylinders represent *Sodalis* that colonize the reproductive organs, midgut, and hemolymph. The light blue corkscrews represent *Spiroplasma* in the reproductive tissues, midgut, and hemolymph in the *palpalis* group of tsetse species.

Four heritable endosymbiotic microbes, *Wigglesworthia glossinidia*, *Sodalis glossinidius, Wolbachia* spp., and *Spiroplasma glossinidia*, have been described across different *Glossina* species and populations (Fig 1). These organisms represent distinct evolutionary associations with their host species, differ in tissue tropism, and influence diverse aspects of their host’s physiology, including metabolism, reproduction and development, and vector competence [7]. *Wigglesworthia*, an obligate mutualist found in all tsetse flies [8], provides its host with essential B vitamins, and mediates the development of a functional immune system during larval development [9,10]. Endosymbiotic *Sodalis* and *Wolbachia* are distributed heterogeneously in some populations of several *Glossina* species. Commensal *Sodalis* may impact tsetse’s longevity and susceptibility to infection with trypanosomes [7], while parasitic *Wolbachia* induces cytoplasmic incompatibility in the fly [11].

Several species within the *palpalis* group of *Glossina* harbor infections with bacteria from the genus *Spiroplasma* [12]. *Spiroplasma* are helical, cell wall‐less Mollicutes that colonize a wide range of arthropod hosts, most commonly insects [13–19]. In these insect systems, *Spiroplasma* exhibit diverse endosymbiotic phenotypes, including protective effects against parasitic wasps and nematodes [20–22], reproductive manipulations that induce male-killing [23,24], and cytoplasmic incompatibility [25]. In the tsetse fly *Glossina fuscipes fuscipes* (*Gff*), functional studies indicate that the *Spiroplasma glossinidia* strain *s*Gff confers both beneficial and detrimental effects on host physiology, including increased resistance to trypanosome infections [26] and reduced host fecundity [27,28].

Here, we provide an overview of *Spiroplasma* infection prevalence in natural tsetse populations, mechanisms of vertical transmission, and physiological interactions between *s*Gff and its tsetse host. We highlight emerging genomic and transcriptomic features that reflect host-endosymbiont metabolic interdependence and examine how *s*Gff infection shapes host metabolic homeostasis, reproductive physiology, and vector competence. Finally, we outline key priorities for advancing the study of the *Gff*-*s*Gff symbiosis, including the identification and functional validation of pathways that induce pathogen resistance, changes in fly mating behavior and physiology, and imperfect bacterial transmission. We conclude by discussing how insights into *Gff*-*s*Gff interactions can inform novel parasite transmission-blocking strategies and identify key priorities for future research efforts  (Box 1).

## *s*Gff infection prevalence in natural populations

**Fig 2:**
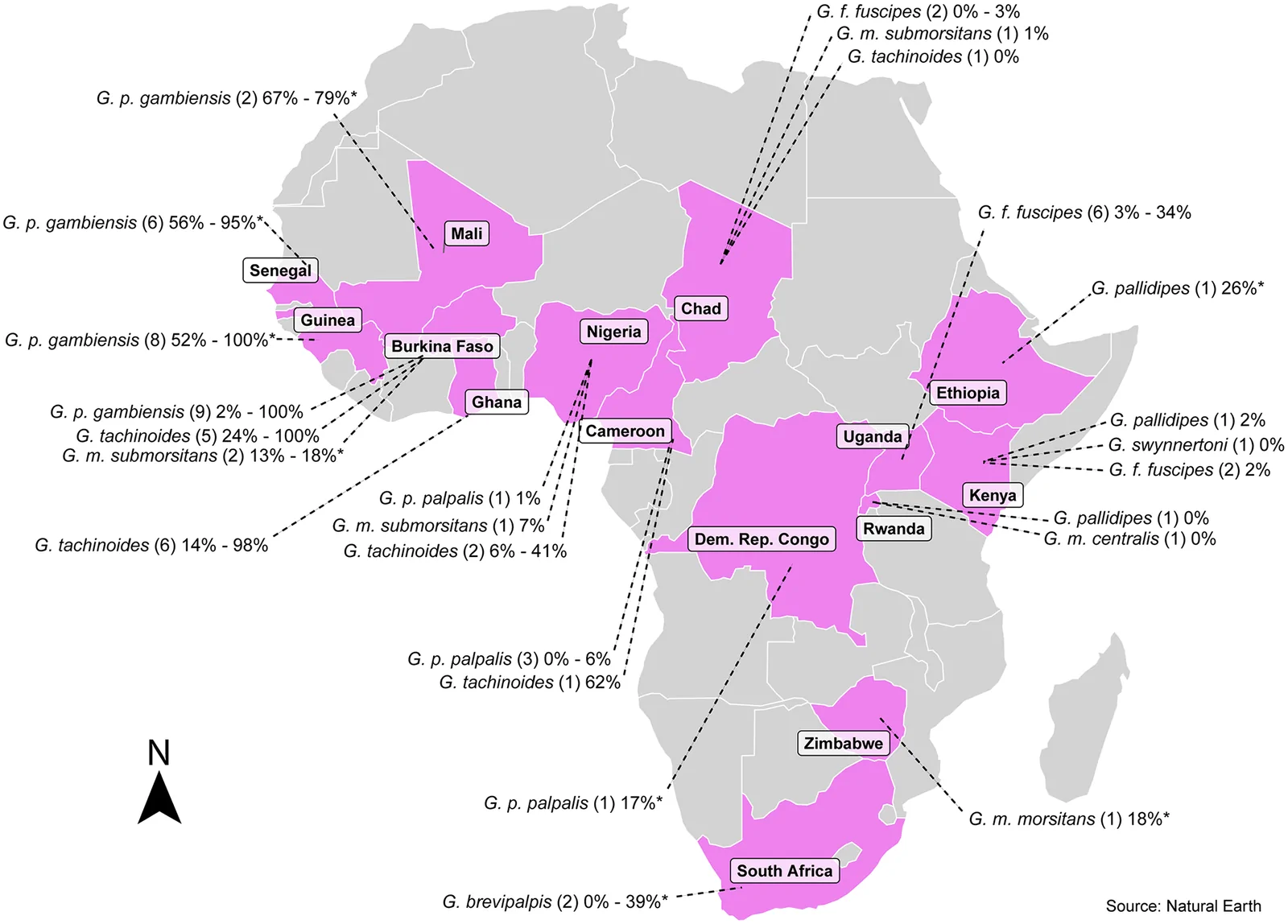
Map of Africa detailing the *Spiroplasma* prevalence data reported in the literature. For each country surveyed, the map shows the range of reported *Spiroplasma* prevalence for each tsetse species and the number of populations surveyed in parenthesis. The * denotes populations where sequence verification of the PCR-amplification product did not occur. The metadata used to populate the map, including the number of surveyed individuals and their associated references, is presented in S1 Table. Countries highlighted in pink are the ones where relevant data can be found in the literature. The map was made using a public domain base layer from Natural Earth (http://www.naturalearthdata.com/about/terms-of-use/).

Field surveys conducted across sub-Saharan Africa indicate that *Spiroplasma* infections are most prominent in tsetse species within the *palpalis* group, including *Gff*, *G. palpalis palpalis*, *G. palpalis gambiensis*, and *G. tachinoides* (Fig 2) [12,26,29–39]. These field surveys further indicate that *Spiroplasma* infections are unevenly distributed across geographic regions, with prevalence ranging from low to moderate. This variance in infection rates could be the result of local tsetse population structure and/or ecological variables. Whether the infections reported in different species and populations represent *s*Gff or a new *Spiroplasma* taxa is currently unknown.

*Spiroplasma* infection prevalence analyses performed by different research groups must employ standardized methods. As such, the community should utilize a common set of *Spiroplasma*-specific PCR primer sets or confirm the putative amplicons by sequencing. This is because a commonly used 16S rDNA primer set that was previously thought to only amplify *Spiroplasma* DNA generated false positives, particularly in tsetse species outside of the *palpalis* group [29]. Several *Spiroplasma*-specific unique genes are promising candidates, including *Spiralin* [40] and cytoskeletal motor fibril protein (*Fib*) [41].

Uganda represents one of the best surveyed countries for *s*Gff prevalence in *Gff* where infection frequencies among genetically differentiated populations are strongly associated with geographic origin [26]. The highest *s*Gff prevalence (5–34%) occurs in populations along the Albert Nile and Achwa River watersheds in the country’s northwest. Across this region, infection frequencies are generally stable over time and space, although seasonal variation is evident, with higher prevalence observed during the intermediate season compared to the wet season [26]. No consistent association has been found between *s*Gff infection and host genetic background or sex, suggesting that local ecological factors rather than host genotype are the primary drivers of infection dynamics in Ugandan *Gff* populations [26].

The apparent affinity of *Spiroplasma* for *palpalis* group species suggests historical host restriction, ecological barriers to horizontal transmission, and/or lineage-specific physiological compatibility between *Spiroplasma* and *palpalis* group hosts. Interestingly, prevalence surveys of *Wolbachia* in *Glossina* find this bacterium to be predominantly associated with tsetse species within the *morsitans* group [42], suggesting that group-specific symbiont partitioning within the genus *Glossina* may occur. It remains to be seen whether competitive exclusion among vertically transmitted symbionts contributes to this distribution, whereby microbial communities influence the establishment and persistence of other endosymbionts [43,44]. In this regard, it is important to investigate within-host dynamics of the broader microbiota composition in both laboratory and field tsetse populations infected with *Spiroplasma* compared to uninfected counterparts.

## *s*Gff tissue tropism and transmission biology

In the IAEA Insect Pest Control Laboratory (IPCL) maintained line of *Gff,* derived from a founder population collected in 1986 from the Central African Republic [45], *s*Gff infection prevalence persists between 50% and 60% [46]. This stable intermediate frequency indicates that *s*Gff exhibits imperfect vertical transmission and/or infection-associated fitness costs that prevent fixation within the colony. *s*Gff is detected primarily in female and male reproductive tissues, consistent with its vertical transmission [12,46]. However, the bacterium is also detected in other tissues, including hemolymph, legs, and gut, indicating a systemic infection [12,47,48]. This broad tissue distribution may facilitate maternal and potentially paternal or horizontal transmission routes, while also providing opportunities for broad host-symbiont interactions that influence metabolic and immune pathways.

Vertical transmission from mother to offspring was consistently high but incomplete, with lower detection in pupae than in adult offspring (67% versus 86% when both parents were infected; 44% versus 97% when only mothers were infected), likely reflecting stage-specific differences in bacterial density and reduced PCR sensitivity at low titers [27]. The same study also provided evidence of paternal *s*Gff transmission. In limited crosses between infected fathers and uninfected mothers, *s*Gff was detected in approximately 44% of offspring [27].

During mating, male tsetse transfer seminal fluid and spermatozoa to the female. Once in the female’s uterus, this mixture forms into a proteinaceous spermatophore that facilitates the successful transfer of the spermatozoa into the spermatheca organ where they are stored long-term for use in multiple gonotrophic cycles. In another study, *s*Gff DNA was detected in the spermatophore and then in the spermathecae of uninfected females mated with infected males, which could have only originated from the male spermatophore [48]. Together, these findings support predominantly maternal transmission of *s*Gff, with additional paternal contributions that may influence symbiont persistence in natural populations.

## In vitro cultivation of *s*Gff

Recently, a new in vitro cultivation system was developed for *s*Gff using hemolymph-derived *s*Gff cells (collected from colonized *Gff*) grown in supplemented Barbour-Stoenner-Kelly H (BSK-H) medium [47]. Characterization of growth kinetics using fluorescent microscopy and qPCR revealed that newly inoculated *s*Gff cultures undergo two distinct phases of exponential growth separated by an intermediate plateau phase. The doubling time of *s*Gff in newly inoculated cultures is 40 hours during the first exponential phase and extends to 85 hours during the second exponential phase. However, these plateau phases are not observed in routinely passaged cultures. Cultures remained viable for >12 months with routine passaging and retained the capacity to establish infection when injected into naive *Gff* [47]. It remains to be determined whether cultured *s*Gff can be experimentally transinfected into novel tsetse host species by microinjection or membrane feeding assays. Such studies would enable testing of fitness costs, tissue tropism, and trypanosome transmission dynamics in tsetse species beyond *Gff*. Importantly, the ability to cultivate *s*Gff in vitro provides a platform to genetically modify the bacterium. Introducing stable, visible reporter markers into *s*Gff would enable tracking of bacterial colonization, tissue distribution, and vertical transmission routes in naive hosts. Additionally, targeted knockdown of genes predicted to play key roles in the tsetse-*Spiroplasma* symbiosis would enable functional characterization of their contribution to host interaction and transmission.

## Insights gained from the *s*Gff genome

### Genome comparisons of multiple sGff strains

The reference genome for the in vitro cultured *s*Gff was assembled using Oxford Nanopore long reads and polished with Illumina short reads (Table 1) [47]. The closed *s*Gff genome is highly dynamic, with more than 40% of the encoded genes categorized as mobile genetic elements (MGEs), consisting primarily of prophages, insertion sequence elements, and integrative conjugative elements. These MGEs likely drive structural rearrangements and gene gain and loss, thus leading to genomic plasticity and rapid diversification [49].

**Table 1:** Genome statistics for the assembled *S. glossinidia* strain *s*Gff.

NCBI RefSeq	GCF_049669535.1
Chromosome size	1.49 mb
Number of plasmids	4
Plasmid sizes	16 kb, 14 kb, 13 kb, 6 kb
BUSCO score	C:97.6% [S:97.6%,D:0.0%]
GC percent	27.20%
Genes	1,829
Protein coding genes	1,687
Pseudo genes	104
tRNA	32
rRNA	3
ncRNA	3
*s*Gff isolated from a single IAEA Gff:	GCF_049949145.1
*s*Gff isolated from a single Ugandan field-captured Gff:	GCF_049949155.1

Two additional complete *s*Gff genomes were generated: one from a single IAEA IPCL colony female *Gff* and one from a single female *Gff* collected in Northwest Uganda [47]. As expected, the genomes from in vitro culture and IPCL *Gff* line were nearly identical, differing by only three SNPs and a duplication of a prophage element. In contrast, the genome derived from field-collected *Gff* exhibited extensive divergence relative to the in vitro *s*Gff, including 550 SNPs, variation in gene content, five prophage duplications, and one prophage translocation [47]. This divergence between laboratory and field-derived genomes suggests substantial genomic variation in natural *s*Gff populations.

**Fig 3:**
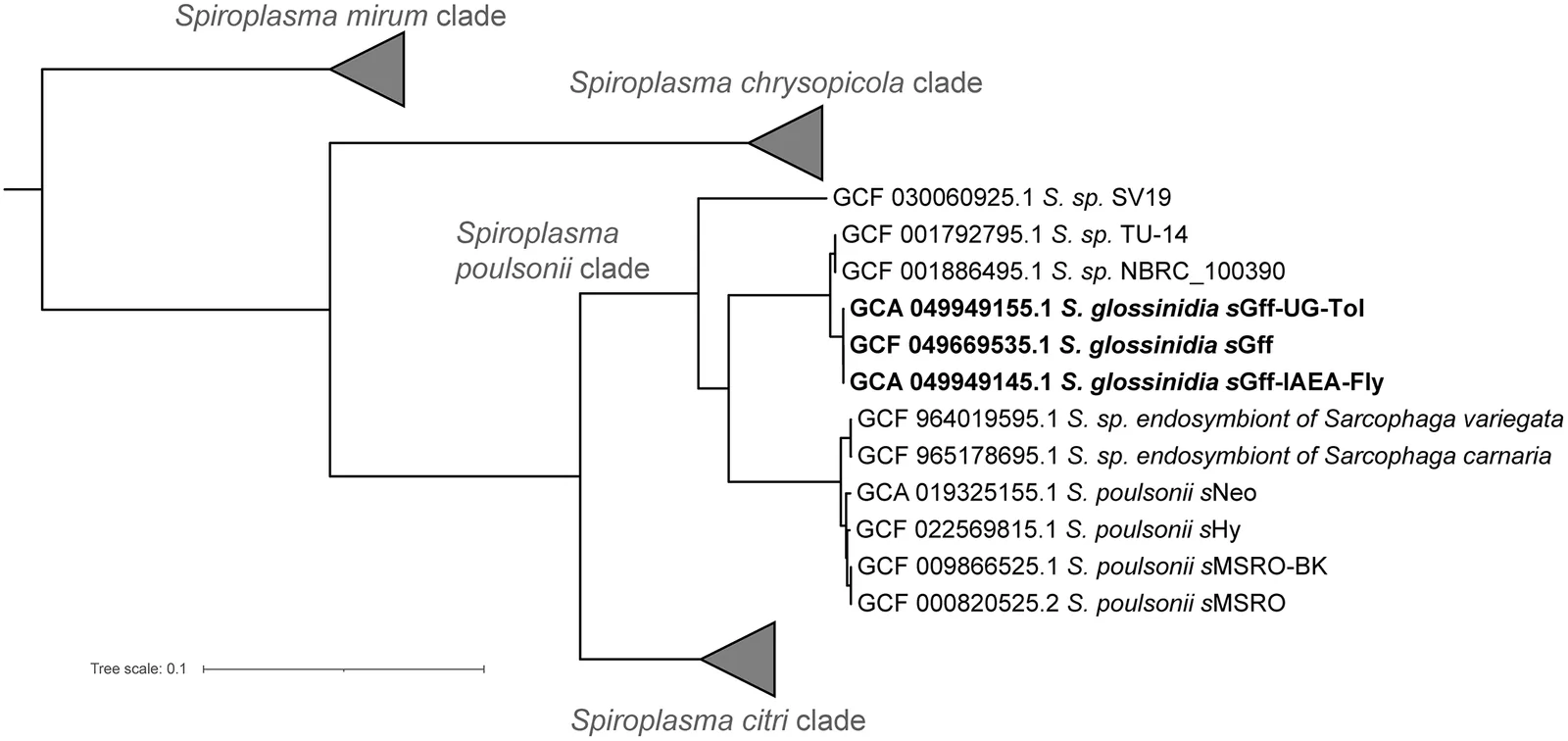
A maximum likelihood phylogeny highlighting *s*Gff’s relationship to other members of the *S. poulsonii* clade and to the *S. mirum*, *S. chrysopicola*, and *S. citri* clades. The phylogeny was constructed using RAxML-NG from an alignment of ~100 single-copy orthologous genes shared with >95% of the tree taxa. Bootstrap support for each node is 100/100. The three *s*Gff isolates form their own clade and are sister to the clade comprised of *S. sp.* TU-14, and *S. sp.* NBRC_100390, two taxa with largely unknown biology.

A phylogeny with other publicly available *Spiroplasma* genomes placed *s*Gff within the *S. poulsonii* clade (Fig 3) [47]. This clade is of particular interest because many of its members induce strong host fitness effects [50]. The two taxa most closely related to *s*Gff are *Spiroplasma sp. s*TU-14 and *Spiroplasma sp. s*NBRC_100390. Beyond their close phylogenetic relationship to *s*Gff, little is known about their biological context [51], including natural host range, ecological distribution, transmission dynamics, and phenotypic effects on hosts.

### *Metabolic capacity predicted from* the *sGff putative proteome*

The *s*Gff genome encodes a highly reduced metabolic repertoire, suggesting a strong dependance on its host’s nutritional landscape [47]. Given that tsetse flies subsist exclusively on vertebrate blood, a diet deficient in several essential nutrients, *s*Gff may also rely on metabolites provisioned by the obligate symbiont *Wigglesworthia,* including B-vitamins [10]. Metabolic interdependence among the fly, its endosymbionts, and trypanosomes is well documented in the tsetse system [52–56].

Based on its putative proteome, *s*Gff energy metabolism appears to rely primarily on glycolysis, utilizing glucose and fructose as major carbohydrate substrates [47]. Like other *Spiroplasma* species, *s*Gff has minimal capacity for *de novo* lipid biosynthesis. Instead, like other *Spiroplasma* species, *s*Gff likely incorporates host-derived fatty acids and sterols directly into its cell membrane [57]. However, *s*Gff retains crucial lipid-modifying functions, particularly the ability to synthesize cardiolipin from host-derived diacylglycerols (DAGs), consistent with the experimentally validated pathway in *S. poulsonii* [58].

### Symbiosis and pathogenesis-related genes

The *s*Gff genome does not encode genes that, in other *Spiroplasma*, are known to induce pathogenic phenotypes such as male killing [23,24] or cytoplasmic incompatibility [25]. The absence of a male killing phenotype is also supported by the fact that the sex ratio of colonized *Gff* remains balanced across generations [40,59]. However, the genome encodes numerous other factors that may facilitate its symbiotic association with its tsetse host. Among these are 72 predicted lipoproteins, including three Spiralin homologs, which are implicated in host-microbe interactions to ensure vertical transmission in other *Spiroplasma* systems [60]. Another group of putative products include six adhesion-related proteins, most of which are plasmid-encoded and are predicted to contribute to host cell adhesion [61] and/or invasion [61].

The *s*Gff genome also encodes several putative toxins, with orthologues that target parasites in other insect systems [62]. Notably, it contains two glycerol-3-phosphatase (*glpO*) homologs. In other Mollicutes, *glpO* can produce reactive oxygen species (ROS) and functions as a major virulence factor in *S. taiwanense*, *S. poulsonii* MSRO, and *Mycoplasma mycoides* [63–65]. Finally, the *s*Gff genome encodes two ribosome-inactivating proteins (RIPs) that are thought to be target-specific [66] and act by depurinating the sarcin-ricin loop of 28S rRNA, thereby blocking translation initiation. This results in inhibition of protein synthesis and ultimately cell death [67]. *Spiroplasma* RIP toxins protect *Drosophila* hosts from parasitoid wasps and parasitic nematodes [20,21,68], and while not *Spiroplasma-*derived, RIP toxins from plants were shown to inactivate ribosomes in vitro from *Trypanosoma brucei rhodesiense* [69]. Further characterization of these candidate toxins is necessary to better understand if they could be involved in *s*Gff interactions with its tsetse host, other endosymbionts, and/or associated pathogens.

### *sGff in vivo and* in vitro *gene expression*

Comparison of transcriptomes from *s*Gff residing in host hemolymph versus *s*Gff grown in vitro identified 41 differentially expressed (DE) genes [47]. Of these DE transcripts, 12 were uniquely upregulated in hemolymph-derived *s*Gff relative to in vitro *s*Gff, including a fructose-specific phosphotransferase system (PTS) transporter, five metabolism-related genes, two translation-related genes, and genes associated with detoxification, repair, and stress responses. Conversely, 29 transcripts were uniquely downregulated in hemolymph-derived *s*Gff relative to in vitro *s*Gff, including a glucose-specific PTS transporter, six metabolism-related genes, two prophage-related genes, two lipoproteins, a DNA polymerase, and a serine protease. Together, these patterns indicate that *s*Gff modulates gene expression across environments and suggest that its physiology may differ across host tissues and immune contexts, warranting further study.

## *s*Gff effects on tsetse biological traits

**Fig 4:**
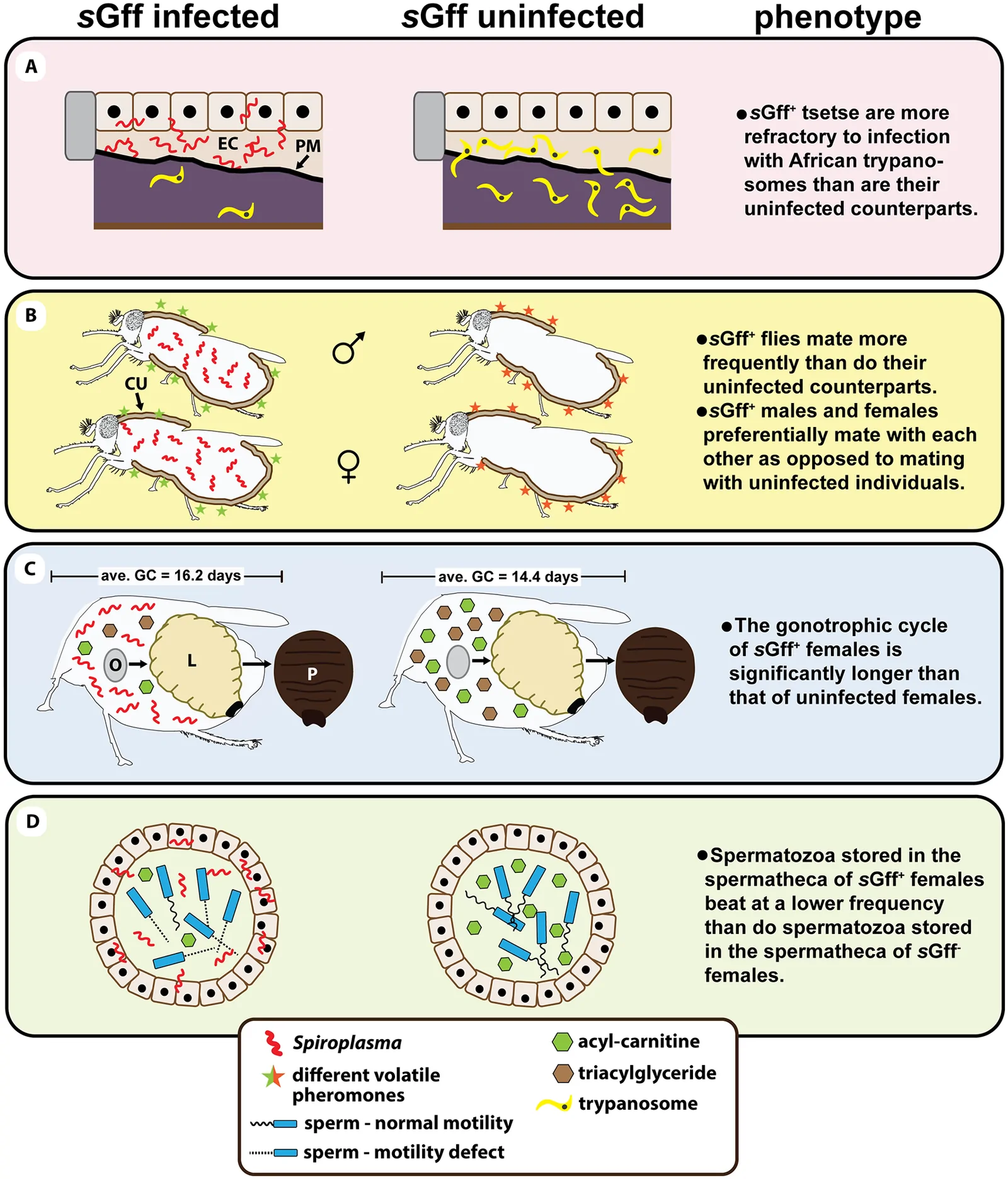
*s*Gff infection effects on tsetse host physiology. **(A)** *s*Gff infection can be beneficial to *Gff*, as flies that house the bacterium are more resistant to infection with parasitic trypanosomes than are their counterparts that do not house the bacterium. The mechanism that underlies this phenotype could result from (1) enhanced basal *Gff* trypanocidal immune responses, (2) production of trypanocidal compounds by *s*Gff, and/or (3) competition with trypanosomes for limited but essential host nutrients. **(B)** *s*Gff infection impacts mating behaviors [e.g., frequency and mate choice (i.e., assortative mating)] of male and female *Gff*. These altered mating behaviors may reflect *s*Gff-induced changes in fly cuticular hydrocarbons, some of which function as pheromones. **(C)** *s*Gff and mated female *Gff* also compete for another class of lipids, triacylglycerides (TAGs). TAGs are the most abundant lipids found in tsetse milk, the sole source of nourishment for developing intrauterine larvae. *s*Gff-mediated depletion of circulating TAG deprives the larvae of this crucial nutrient, thus delaying its development, lengthening the gonotrophic cycle, and decreasing the fly’s overall fecundity. **(D)** When mated females are infected with *s*Gff, spermatozoa stored in their spermatheca exhibit a motility defect. This phenotype occurs because *s*Gff consumes circulating and spermatheca-borne acylcarnitines, which are lipids that both the bacterium and stored spermatozoa require to maintain their physiological homeostasis. CU, cuticle; O, oocyte; L, larva; P, pupa; EC, ectoperitrophic space [trypanosomes must cross tsetse’s peritrophic matrix (PM) and establish an infection in the fly’s ectoperitrophic space to be transmitted to a new vertebrate host].

Microbial endosymbionts fulfill diverse and often essential functions in their insect hosts, including nutrient provisioning, regulation of digestion and reproduction, detoxification, maintenance of immune homeostasis, and protection against pathogens. Endosymbionts can also influence host behavior, including mating choice, which over evolutionary time, can contribute to reproductive isolation and speciation [70–73]. Below, and summarized graphically in Fig 4, we detail current knowledge of *s*Gff-tsetse interactions, focusing on mating behavior, fecundity, and susceptibility to infection with pathogenic African trypanosomes.

### Impact on host vector competence

Multiple studies in tsetse report a negative association between *s*Gff infection and susceptibility to African trypanosomes, both under controlled laboratory conditions and across several field sites [26,29]. When laboratory-reared *Gff* with about 50% *s*Gff infection prevalence were offered a trypanosome infectious blood meal, significantly fewer *s*Gff-infected individuals (18%) became infected with parasites than did their *s*Gff-uninfected counterparts (46%) [26]. In northwest Uganda, where *s*Gff is highly prevalent in *Gff* populations, only 2% of flies were coinfected with both *s*Gff and trypanosomes, compared to a 10% trypanosome infection prevalence among *s*Gff-uninfected flies [26]. Similarly, surveys of *G. tachinoides* across multiple sites revealed lower trypanosome infection prevalence among *Spiroplasma*-infected flies compared to uninfected individuals [29]. Collectively, these laboratory and field observations support a consistent negative association between *Spiroplasma* infection and trypanosome infection across at least two tsetse species.

### Impact on mating behavior

Mating experiments suggest that *s*Gff infection may influence tsetse mating behavior. Female choice assays have yielded mixed results: one study found females, regardless of their *s*Gff infection status, preferentially mated with uninfected males over infected males [46], whereas another study reported higher mating frequency among *s*Gff-infected flies of both sexes [48]. The latter study also provided evidence for assortative mating [choosing of a partner based on a specific trait(s)], which in this case could reflect mate choice based on shared *s*Gff infection status [48]. Together, these findings suggest that *s*Gff may manipulate host mating behavior to facilitate its transmission and spread within tsetse populations.

The underlying physiological mechanisms for *s*Gff-induced behaviors are currently unknown, but symbiont-induced changes in host chemical signaling represent a plausible explanation. Cuticular hydrocarbons, key mediators of insect mating behavior [74,75], can be altered by symbiotic bacteria, as demonstrated by *Wolbachia-*induced changes in *G. m. morsitans* [76]. Because these compounds stand to influence tsetse mating behaviors, further research is required to determine whether *s*Gff similarly alters cuticular hydrocarbon composition and/or the fly’s behavioral responses.

### Impact on host metabolism

As an endosymbiont, *s*Gff relies entirely on host-derived nutrients, with lipids appearing particularly critical for both *Spiroplasma* and host metabolic homeostasis. Experimental evidence indicates that *s*Gff hijacks tsetse-derived lipids as infected females have lower circulating triacylglycerides (TAGs) in their hemolymph. This finding mirrors *Drosophila* infected with *S. poulsonii* [58], where the bacterium captures host DAG species for production of cardiolipin, thereby reducing the fly’s circulating DAG and TAG reserves.

In addition to consuming TAGs, *s*Gff utilizes acylcarnitines to support its metabolic homeostasis. The insect fat body and gut produce acylcarnitines to fuel energy-intensive physiological processes, including nutrient metabolism and nutrient mobilization. These molecules circulate in the hemolymph and are transported to metabolically intensive tissues such as flight muscles [77]. Fat bodies of mated, *s*Gff-infected females produce more acylcarnitines than those of mated, uninfected females. However, despite this enhanced production, acylcarnitines are less abundant in the hemolymph of both laboratory-reared and field-captured *s*Gff-infected *Gff* compared to their uninfected counterparts, suggesting that the bacteria may be consuming these lipids. In support of this theory, suppressing acylcarnitine production via RNAi-mediated knockdown of *Gff* carnitine O-palmitoyltransferase-1 (the enzyme that forms acylcarnitines by conjugating free fatty acids with L-carnitine [78]), results in a significant reduction in *s*Gff density, as measured by16S rRNA transcript abundance. Furthermore, when *s*Gff cells are cultured in medium that lacks palmitic and oleic acids, they assume a coccus-like (as opposed to helical) morphology and fail to replicate. Complementation of these fatty acids back into the growth media rescues the phenotypic defects [28]. Interestingly, palmitic and oleic acids are the fatty acid components of palmitoylcarnitine and oleoylcarnitine, which are highly abundant in *Gff* hemolymph and among the most significantly depleted metabolites in *s*Gff-infected flies [28]. Although the physiological role of palmitic and oleic acid in supporting *s*Gff growth in vitro remains unknown, studies on closely related *Spiroplasma* strains (*S. floricola* and *S. citri*) indicate that Spiralin, which is the most abundant proteinaceous component of bacterial cell membrane, is highly palmitoylated [79]. Spiralin palmitoylation likely anchors the protein to *Spiroplasma’s* cell membrane, enhancing its structural integrity and helping maintain bacterial shape and viability.

### Impact on host reproduction and fecundity

Transcriptomic analyses of reproductive organs from *Gff* captured in natural populations in Uganda revealed that *s*Gff infection is associated with sex-specific changes in gene expression [27]. In females, *s*Gff infection was associated with the upregulation of female-biased genes and downregulation of male-biased genes. Given that sex-biased gene expression can influence sexual phenotypes that mediate fecundity, the functional consequences of these shifts for tsetse reproduction are important. Recent work on *Gff* further identified differential gene expression between *s*Gff-infected and uninfected testes and male accessory glands in colonized flies [80]. Functional analyses of such differentially expressed products would provide further insights into how this endosymbiont impacts its hosts reproduction.

Although *s*Gff reduces trypanosome infection prevalence and presumably the associated metabolic burden, it imposes a reproductive cost on its tsetse host by reducing the fly’s fecundity [27]. This fecundity cost results from a significantly prolonged gonotrophic cycle (GC), with each of the first three cycles analyzed extended by approximately 2 days relative to 10 days in uninfected flies [27]. Because female tsetse flies produce a maximum of 8–10 offspring over their lifespan [5], the lengthened GC could have a substantial negative impact on the population size. In support of this theory, the recent collapse of a *Gff* colony maintained in Bratislava may have been associated with the high *s*Gff infection prevalence noted in this line (>80%) [12], whereas stable laboratory and field *Gff* populations typically maintain *s*Gff prevalence at or below 50% [26,46].

The *s*Gff-induced reduction in tsetse fecundity may arise from two bacterium-induced physiologies. First, TAGs, which *s*Gff depletes from its host’s hemolymph, are the most abundant lipids in tsetse milk [81,82] that serve as the sole nutrient source for developing intrauterine larvae. Competition between *s*Gff and the host for TAGs could reduce their availability for incorporation into the milk, thus depleting its nutritional value and prolonging larval development and GC length. Second, spermatozoa stored in the spermatheca of *s*Gff-infected females beat at significantly lower frequencies than spermatozoa stored in the spermatheca of uninfected females [28], reducing sperm fitness and the reproductive potential of infected females. This phenotype may result, at least in part, from competition between *s*Gff and spermatozoa for acylcarnitines within the spermatophore. As noted above, *s*Gff relies upon tsetse-derived acylcarnitines to maintain its metabolic homeostasis, and stored tsetse spermatozoa, like their mammalian counterparts, also relies on acylcarnitines as an important source of energy. Lipidomic analyses show that tsetse spermatheca produce five acylcarnitines, four of which are produced in significantly higher quantities following mating [28]. When acylcarnitine production is experimentally inhibited in mated females, stored spermatozoa become less motile, the size of the stored spermatozoa bundle shrinks, and is eventually resorbed, leading to reproductive sterility [28]. Taken together, these findings indicate that *s*Gff competes with its tsetse host for critical TAGs and acylcarnitines. By sequestering these critical lipids, *s*Gff deprives tsetse of nutrients that support larval development and proper spermatozoa function, thus significantly reducing host fecundity.

## Potential mechanisms by which *s*Gff influences host vector competence

To gain insight into *s*Gff influences on host vector competence, midgut transcriptomes of uninfected *Gff* (no *s*Gff and no trypanosomes) were compared with those derived from flies infected with either *s*Gff or trypanosomes [83]. Only 69 genes were differentially expressed in response to *s*Gff, while 989 changed in response to trypanosomes, indicating a substantially weaker host transcriptional response to the bacterium.

Among the genes responsive to both microbes were several associated with development and structural integrity of the fly’s peritrophic matrix (PM) (four *peritrophins*, *chitin synthase* 2 (*chs*2) and a chitin-binding protein), as well as PM turnover (chitinase 2 and chitin deacetylase 8). Because multiple studies have reported that PM disruption facilitates parasite colonization of tsetse’s midgut [83–85], the upregulation of PM-associated genes in *s*Gff-infected flies may represent a compensatory response that strengthens the PM barrier’s function, thus limiting parasite establishment. In addition to PM-related genes, several transcripts that encode immunity-related genes were induced by both *s*Gff and trypanosomes, including *C-type lectin*, *mucin-5AC*, and *nitric oxide synthase*. These factors are implicated in anti-trypanosome immune responses in *Gmm,* with nitric oxide exhibiting potent trypanocidal activity [86]. Elevated expression of these immune factors in *s*Gff*-*infected flies may therefore contribute to their enhanced resistance to trypanosome infection. Notably, trypanosome challenge also activates canonical innate immune pathways, including the Immune Deficiency (Imd) pathway, which leads to the production of antimicrobial peptides with documented trypanocidal activity [86]. In contrast, these canonical pathways are not detectably activated in response to *s*Gff, indicating that *Spiroplasma* does not trigger a classical innate immune response in tsetse.

In addition, 31 genes responded uniquely to *s*Gff infection, many involved with metabolic processes, particularly lipid biosynthesis, including fatty acid synthases (FAS), fatty acyl-CoA reductases, and acyl-CoA binding proteins. Several of these transcripts, highly expressed under basal conditions, were strongly downregulated in *s*Gff-infected flies. Reduced lipid biosynthetic capacity in the midgut could have important implications for both host metabolic homeostasis and parasite development. Consistent with this hypothesis, flies infected with *s*Gff and/or trypanosomes present with significantly reduced TAG levels [83], suggesting diminished host lipid synthesis and/or increased microbial utilization. These findings suggest that competition for host lipids may contribute to the refractoriness phenotype observed in *s*Gff-infected flies, highlighting the importance of host–symbiont–parasite metabolic interplay in vector competence.

In addition to host-induced immune molecules, *s*Gff products could also impact trypanosome colonization in the midguts where both microbes live in close proximity. As discussed in the section titled ‘*Symbiosis and pathogenesis related genes*’, one such product would be the putative RIP proteins, which are detected in the transcriptomes of *s*Gff-infected flies [47].

## Conclusions

Here we present an overview of the current knowledge on the endosymbiont *S. glossinidia* that preferentially infects *palpalis* group tsetse species across the African continent (Fig 2, Box 2). Studies to date indicate that housing *Spiroplasma* represents a double-edged sword for its tsetse host. Infections with the bacterium significantly reduce fly fecundity. However, housing the bacterium can also significantly increase fly fitness, as trypanosome infection is metabolically costly and adversely impacts fly fecundity. These apparent trade-offs indicate that *s*Gff could be a promising candidate for biocontrol applications where both deleterious fitness effects and trypanosome resistance phenotypes are desired outcomes. In Box 3, we highlight several priority research areas that stand to advance fundamental knowledge on the tsetse-*Spiroplasma* symbiosis such that it may be experimentally exploited to reduce tsetse-borne disease.

**Box 1. Key papers**

1. Doudoumis, V., Blow, F., Saridaki, A., Augustinos, A., Dyer, N.A., Goodhead, I., Solano, P., Rayaisse, J.B., Takac, P., Mekonnen, S., Parker, A.G., Abd-Alla, A.M.M., Darby, A., Bourtzis, K Tsiamis, G., 2017. Challenging the *Wigglesworthia*, *Sodalis*, *Wolbachia* symbiosis dogma in tsetse flies: *Spiroplasma* is present in both laboratory and natural populations. Sci Rep 7:4699
2. Schneider, D.I., Saarman, N., Onyango, M.G., Hyseni, C., Opiro, R., Echodu, R., O’Neill, M., Bloch, D., Vigneron, A., Johnson, T.J., Dion, K., Weiss, B.L., Opiyo, E., Caccone, A., Aksoy, S., 2019. Spatio-temporal distribution of *Spiroplasma* infections in the tsetse fly (*Glossina fuscipes fuscipes*) in northern Uganda. PLoS Negl Trop Dis 13, e0007340.
3. Son, J.H., Weiss, B.L., Schneider, D.I., Dera, K.M., Gstottenmayer, F., Opiro, R., Echodu, R., Saarman, N.P., Attardo, G.M., Onyango, M., Abd-Alla, A.M.M., Aksoy, S., 2021. Infection with endosymbiotic *Spiroplasma* disrupts tsetse (*Glossina fuscipes fuscipes*) metabolic and reproductive homeostasis. PLoS Pathog 17, e1009539.
4. Awuoche, E.O., Smallenberger, G., Bruzzese, D.L., Orfano, A., Weiss, B.L., Aksoy, S., 2025. *Spiroplasma* endosymbiont reduction of host lipid synthesis and Stomoxyn-like peptide contribute to trypanosome resistance in the tsetse fly *Glossina fuscipes*. PLoS Pathog 21, e1012692.
5. Bruzzese, D.J., Gstottenmayer, F., Weiss, B.L., Khalil, H., Mach, R.L., Abd-Alla, A.M.M., Aksoy, S., 2025. Comparative genomics and transcriptomics of the *Spiroplasma glossinidia* strain *s*Gff reveal insights into host interaction and trypanosome resistance in *Glossina fuscipes fuscipes*. BMC Genomics 26, 1131.
6. Weiss, B.L., Gstottenmayer, F., Awuoche, E., Smallenberger, G.M., Attardo, G.M., Scolari, F., Koch, R.T., Bruzzese, D.J., Echodu, R., Opiro, R., Malacrida, A., Abd-Alla, A. M. M., Aksoy, S., 2025. Endosymbiont hijacking of acylcarnitines regulates insect vector fecundity by suppressing the viability of stored sperm. PLoS Genet 21, e1011974.

**Box 2. Key learning points**

1. Imperfect maternal and/or paternal transmission to progeny, combined with infection-associated fitness costs, may drive heterogeneous *Spiroplasma* infection prevalences within laboratory and natural tsetse host populations.
2. The ability to culture *s*Gff provides opportunities to develop genetically modified strains and to further understand *Spiroplasma* physiology and host–symbiont–trypanosome infection dynamics. These advances could transform the tsetse-*Spiroplasma* system into a powerful experimental model for understanding host-symbiont dynamics and for developing new vector control methods.
3. Comparative genomic analysis of distinct *s*Gff strains reveals extensive genomic plasticity despite high overall nucleotide identity. More than 40% of the genome consists of mobile genetic elements, including prophages, insertion sequences, and integrative conjugative elements, which may facilitate rapid genome evolution and adaptation in *s*Gff*.*
4. *s*Gff infection extends tsetse’s gonotrophic cycle and reduces the motility, and thus fitness, of stored spermatozoa. These effects may result from competition for tsetse-derived lipids, which are essential for maintenance of the fly’s reproductive physiology.
5. *s*Gff-mediated reduction in tsetse vector competence may result in part from bacterial activation of fly immune molecules (e.g., nitric oxide), which likely renders the tsetse gut environment inhospitable to parasites. *s*Gff may also compete with trypanosomes for limited nutrients that both microbes require to remain viable in the fly.

**Box 3. Future research directions**

1. **Characterize *Spiroplasma* transmission biology.** Understanding the mechanism(s) and efficiency of *Spiroplasma* transmission is critical, as infection prevalence remains heterogeneous in both laboratory and field populations. Studies are needed to identify bottlenecks that drive imperfect maternal and/or paternal transmission. In addition, investigating *Spiroplasma* tissue tropism across the fly lifecycle will provide insight into how infections are established and colonization progresses.
2. **Utilize in vitro *Spiroplasma* cultures to investigate host-symbiont-parasite interactions.** The ability to culture *Spiroplasma* will enable functional studies on host-symbiont-trypanosome interactions (e.g., nutrient competition, host immune activation, and toxin production). Transinfecting *Spiroplasma* into different tsetse species will provide insight into how the bacterium and fly interact immunologically and metabolically across host species and shed light on the group-specific distribution of *Spiroplasma* within genus *Glossina*.
3. **Understand *Spiroplasma-*tsetse evolutionary interactions.** Whole-genome sequencing of *Spiroplasma* isolates across diverse tsetse species and geographic populations will provide a framework for understanding natural *Spiroplasma* variation in tsetse and its evolutionary history. Complementary genome-wide association studies comparing infected and uninfected flies could identify host genetic factors underlying the highly focal distribution of *Spiroplasma* infections.
4. **Investigate mechanisms for *Spiroplasma*-mediated tsetse vector competence reduction.** Genomic data suggests that *Spiroplasma* may produce trypanocidal molecules. Additionally, both the bacterium and trypanosomes compete for the limited supply of host nutrients. Experiments are needed to understand symbiont and host factors towards the reduced vector competence presented by *Spiroplasma-*infected flies.
5. **Assess *Spiroplasma* infection impacts on tsetse mating behavior.** *Spiroplasma* infection alters tsetse’s mating behavior. Studies are necessary to investigate this phenotype, including potential changes to the composition and abundance of tsetse cuticular hydrocarbons, which are known to influence the fly’s mating behavior.
6. **Employ *Spiroplasma* in tsetse Sterile Insect Technique (SIT) programs.** Male tsetse flies, irradiated and released into the environment for use in SIT applications, are potential vectors of pathogenic trypanosomes. Studies are needed to determine whether irradiated males can be colonized with *Spiroplasma* in mass-rearing facilities to render them refractory to trypanosome infection prior to release into the field.

## Supporting information

S1 Table Metadata used to populate the map depicted in Fig 2. (XLSX)
